# How are APOE4, changes in body weight, and longevity related? Insights from a causal mediation analysis

**DOI:** 10.3389/fragi.2024.1359202

**Published:** 2024-03-01

**Authors:** Rachel Holmes, Hongzhe Duan, Olivia Bagley, Deqing Wu, Yury Loika, Alexander Kulminski, Anatoliy Yashin, Konstantin Arbeev, Svetlana Ukraintseva

**Affiliations:** Biodemography of Aging Research Unit, Social Science Research Institute, Duke University, Durham, NC, United States

**Keywords:** mediation, longevity, causal inference, *ApoE4*, aging, weight

## Abstract

The ε4 allele of the APOE gene (*APOE4*) is known for its negative association with human longevity; however, the mechanism is unclear. *APOE4* is also linked to changes in body weight, and the latter changes were associated with survival in some studies. Here, we explore the role of aging changes in weight in the connection between *APOE4* and longevity using the causal mediation analysis (CMA) approach to uncover the mechanisms of genetic associations. Using the Health and Retirement Study (HRS) data, we tested a hypothesis of whether the association of *APOE4* with reduced survival to age 85+ is mediated by key characteristics of age trajectories of weight, such as the age at reaching peak values and the slope of the decline in weight afterward. Mediation effects were evaluated by the total effect (TE), natural indirect effect, and percentage mediated. The controlled direct effect and natural direct effect are also reported. The CMA results suggest that *APOE4* carriers have 19%–22% (TE *p* = 0.020–0.039) lower chances of surviving to age 85 and beyond, in part, because they reach peak values of weight at younger ages, and their weight declines faster afterward compared to non-carriers. This finding is in line with the idea that the detrimental effect of *APOE4* on longevity is, in part, related to the accelerated physical aging of ε4 carriers.

## 1 Introduction

The ε4 allele of the *APOE* gene (*APOE4*) is known for its negative association with human longevity (Arbeev et al., 2011; Kulminski et al., 2011; Arbeev et al., 2012; Kulminski et al., 2014; Garatachea et al., 2015; Zeng et al., 2016; Yashin et al., 2018a; Yashin et al., 2018b; Abondio et al., 2019; Gurinovich et al., 2019; Kulminski et al., 2019; Santovito, Galli, and Ruberto, 2019; Sebastiani et al., 2019; Kulminski et al., 2022; Arbeev et al., 2023), although the mechanism is not well understood. Certain metabolic phenotypes (e.g., diabetes and serum lipids) were proposed as potential mediators; however, their mediating role in the association of *APOE4* with longevity was not confirmed (Noordam et al., 2016). *APOE4* was also linked to a lower weight and body mass index (BMI) and its changes with age (Bäckman et al., 2015; Bell et al., 2017; Blautzik et al., 2018; Kulminski et al., 2019; Ando et al., 2022; Ukraintseva et al., 2024). We recently demonstrated, using HRS and FHS data, that *APOE4* carriers had lower weight than non-carriers starting approximately at age 65 and reached the maximum weight at younger ages compared to non-carriers, as well as declined in weight faster than non-carriers (Ukraintseva, et al., 2024).

Weight/BMI and their changes later in life were associated with survival in older ages (Lee et al., 2001; Køster-Rasmussen et al., 2016). In a study of post-menopausal women, it was found that weight loss, especially if unintentional, over a period of three to ten years, was associated with significantly lower chances of longevity (survival to ages over 90) compared to women with stable weights (Shadyab et al., 2023). Previously, we showed that entire age trajectories of weight/BMI can significantly differ between longer- and shorter-lived individuals (Yashin et al., 2013; Yashin et al., 2016); the shorter-lived individuals reached maximum values of weight/BMI and started to decline earlier in their lives compared to the longest living individuals. Trajectories of aging-related changes in weight may also differ between longer- and shorter-lived strains of laboratory rodents such that the longer-lived strains typically reach maximum values of weight later in their lives (Turturro et al., 1999). The decline in weight at older ages may reflect the physical and physiological changes in the body that occur with aging, such as the loss of muscle mass (sarcopenia) and reduction in food absorption, among others (Bales and Ritchie, 2002; Volpi, Nazemi, and Fujita, 2004; Woods, Iuliano-Burns, and Walker, 2011; Curtis et al., 2023). If an aging person reaches their peak weight at a younger age and subsequently experiences faster weight decline than their age peers, this could be an indicator of accelerated physical aging, which, in turn, may contribute to reduced chances of extreme longevity (Yashin et al., 2010; Yashin et al., 2013; Yashin et al., 2016).

This and other evidence indicate a possibility that some of the *APOE4* effects on longevity might be attributed to the dynamics of aging-related changes in weight/BMI. Here, we explore the causal relationships between *APOE4* carrier status, key characteristics of mean age trajectories of body weight (such as age at reaching maximum and the slope of decline), and survival to the oldest old age (85+), applying the causal mediation analysis (CMA) (Imai, Keele, and Tingley, 2010; Valeri and VanderWeele, 2013) to the Health and Retirement Study (HRS) data. We propose that *APOE4* may influence age trajectories of the weight, and the negative effect of *APOE4* on longevity could, in part, be because it promotes the earlier/faster decline of weight in older adults.

## 2 Materials and methods

### 2.1 Data

For this analysis, Health and Retirement Study data were used; the HRS is sponsored by the National Institute on Aging and is conducted by the University of Michigan. Beginning in 1992, the National Institute on Aging and the University of Michigan began collecting a variety of phenotypic information with a focus on aging and aging-related information on participants aged 50+ in the United States. Since then, it has grown and expanded to about 20,000 participants and is now one of the largest longitudinal studies of those aged 50+ in the United States. The participants are provided with physical informed consent information prior to each interview, and they are required to verbally consent. More details on the study design and sample selection can be found in Sonnega et al. (2014). Phenotype information was derived from the RAND HRS Longitudinal File (version 2016v1), which is an easy-to-use dataset based on the HRS core data. This file was developed at RAND with funding from the National Institute on Aging and the Social Security Administration. It contains a variety of information from the interviews of HRS participants, such as demographics, health, employment, and retirement.

For the present analysis, a subset of participants was selected: those who survived to at least age 80 and those with genotype information for *APOE4*. The *APOE4* status was derived from imputed genotype information obtained from the database of Genotypes and Phenotypes (dbGaP) HRS data (dbGaP Study Accession: phs000428. v2. p2), which includes genotype information for 2.5 million SNPs collected using the Omni2.5 BeadChip. Further details regarding the characteristics of the participants in the entire HRS sample and the analytic sample can be found in Tables 1 and 2; comorbidity information can be found in these tables as well. Furthermore, we tested if there was a significant difference between the incidences of comorbidities among the groups in the mediators. We found there was no significant difference between occurrences of cancer nor cardiovascular disease among the groups for *SlopeW* and *AgeMaxW.*

**TABLE 1:** Characteristics of the entire HRS sample.

HRS	Men		Women		Total	
*APOE4* Carrier	No	Yes	No	Yes	No	Yes
N	4,674	1,678	6,545	2,430	11,219	4,108
Continuous variables: mean (standard deviation)						
*Entry age*	57.86 (7.82)	57.64 (7.20)	56.68 (9.21)	56.17 (8.65)	57.17 (8.68)	56.77 (8.39)
*End of follow-up age*	75.33 (10.44)	75.01 (10.13)	75.14 (11.44)	74.52 (10.96)	75.22 (11.04)	74.72 (10.63)
*Birth cohort*	1,940.68 (11.38)	1,940.82 (11.08)	1,941.26 (12.20)	1,941.89 (11.72)	1,941.02 (11.87)	1,941.45 (11.47)
Dichotomous variables: N (percentage): number and percentage of individuals in group “1”						
*Education, Highschool+*	4,166 (89.38)	1,488 (89.00)	5,902 (90.41)	2,212 (91.33)	10,068 (89.98)	3,700 (90.38)
*Ever smoked*	3,132 (67.41)	1,135 (68.25)	3,139 (48.25)	1,225 (50.72)	6,271 (56.23)	2,360 (57.87)
*AgeMaxW*	562 (37.24)	179 (33.65)	848 (40.57)	239 (32.34)	1,410 (39.18)	418 (32.89)
*SlopeW*	705 (51.31)	225 (46.20)	990 (52.83)	292 (43.00)	1,695 (52.19)	517 (44.34)
*Survival85*	929 (53.27)	315 (47.73)	1,411 (64.84)	444 (57.66)	2,340 (59.69)	759 (53.08)
*Race: Black*	545 (11.67)	319 (19.02)	945 (14.45)	538 (22.15)	1,490 (13.29)	857 (20.87)
*Race: White*	3,442 (73.69)	1,182 (70.48)	4,599 (70.31)	1,619 (66.65)	8,041 (71.71)	2,801 (68.22)
*Cancer*	1,192 (25.50)	439 (26.16)	1,398 (21.36)	524 (21.56)	2,590 (23.09)	963 (23.44)
*Cardiovascular disease*	2,200 (47.07)	774 (46.13)	2,587 (39.53)	990 (40.74)	4,787 (42.67)	1,764 (42.94)

**TABLE 2:** Characteristics of HRS participants in the *APOE4* subsample who lived up to age ≥80.

HRS	Men		Women		Total	
*APOE4* carrier	No	Yes	No	Yes	No	Yes
N	1,655	576	2,331	805	3,986	1,381
Continuous variables: mean (standard deviation)						
*Entry age*	65.41 (7.38)	65.22 (7.32)	65.89 (7.47)	65.13 (7.28)	65.69 (7.44)	65.17 (7.30)
*End of follow-up age*	86.60 (4.72)	86.12 (4.44)	87.48 (5.34)	86.76 (5.06)	87.11 (5.11)	86.49 (4.82)
*Birth cohort*	1,928.69 (5.97)	1,929.08 (5.63)	1,928.32 (6.38)	1,928.95 (6.17)	1,928.48 (6.21)	1,929.00 (5.95)
Dichotomous variables: N (percentage): number and percentage of individuals in group “1”						
*Education, Highschool+*	1,446 (87.37)	488 (84.87)	2,064 (88.55)	722 (89.69)	3,510 (88.06)	1,210 (87.68)
*Ever smoked*	1,121 (68.52)	401 (70.47)	1,001 (43.17)	354 (44.19)	2,122 (53.65)	755 (55.11)
*AgeMaxW*	562 (37.24)	179 (33.65)	848 (40.57)	239 (32.34)	1,410 (3,918)	418 (32.89)
*SlopeW*	705 (51.31)	225 (46.20)	990 (52.83)	292 (43.00)	1,695 (52.19)	517 (44.34)
*Survival85*	929 (79.40)	315 (76.64)	1,411 (86.67)	444 (81.02)	2,340 (83.63)	759 (79.14)
*Race: Black*	143 (8.64)	65 (11.28)	239 (10.25)	125 (15.53)	382 (9.58)	190 (13.76)
*Race: White*	1,374 (83.02)	466 (80.90)	1,868 (80.14)	624 (77.52)	3,242 (81.33)	1,090 (78.93)
*Cancer*	603 (36.44)	210 (36.46)	630 (27.03)	223 (27.70)	1,233 (30.93)	433 (31.35)
*Cardiovascular disease*	1,082 (65.38)	366 (63.54)	1,236 (53.02)	452 (56.15)	2,318 (58.15)	818 (59.23)

### 2.2 Statistical analysis

**FIGURE 1:**
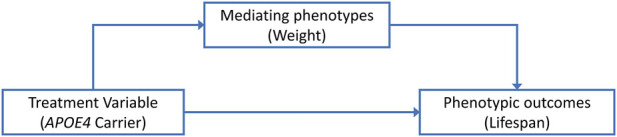
Simplified description of the idea behind a causal mediation analysis (CMA).

In general, the mediation analysis concerns two main pathways: direct and indirect. Illustrated in Figure 1, the direct pathway is seen by the effect of the “treatment” (*APOE4* carrier status in our case) on the outcome. The indirect path is illustrated by the indirect effect of the “treatment” on the outcome, as mediated through the mediator.

Mediation analysis measures the direct and indirect effects illustrated in Figure 1 based on an outcome model and a mediator model (Eq. 1). The general form of these models, respectively, is expressed as follows:

$$\begin{array}{c} outcome=c^{\prime }∙treatment+\beta ∙mediator+\delta_{1}∙covariates,\\ mediator=\alpha ∙treatment+\delta_{2}∙covariates. \end{array}$$ (1)

Specifically, the direct effect is measured by the effect of the treatment on the outcome $c^{\prime }$ , and the indirect effect is measured by αβ . In other words, the total effect (Eq. 2), commonly denoted as c , can be expressed by

$$total\;effect=c^{\prime }+\alpha ∙\beta =c.$$ (2)

Additionally, there exists a measure to determine the amount of mediation, called the percentage mediated (PM), which is calculated by dividing the indirect effect by the total effect (Eq. 3):

$$\frac{\alpha \beta }{c^{\prime }+\alpha \beta }.$$ (3)

This measures the percentage of the effect of the treatment on the outcome that can be attributed to the mediator. In order to claim “complete mediation,” the PM should be at least 80% (Kenny, 2021).

We performed causal mediation analysis, an extension of traditional mediation analysis (TMA) (Baron and David, 1986), in the HRS data analytic sample (see Table 2) using the SAS software application version 9.4. CMA utilizes a counterfactual framework to describe the direct, indirect, and total effect estimates using natural and controlled effects. In this analysis, for example, a counterfactual approach consists of evaluating the chances of survival for each individual at both “treatment” levels even though each individual can only have one “treatment” level (i.e., an individual cannot be a carrier and a non-carrier of *APOE4*). The potential outcome for all individuals is then averaged to calculate the overall potential outcome at each “treatment” level. A variety of potential outcomes at differing mediator and “treatment” levels is used to calculate specific CMA estimates. Additionally, CMA effects are not bound by parametric assumptions, unlike TMA effects (Rijnhart et al., 2021).

*APOE4* status was used as a binary “treatment” variable in the CMA: (1) *APOE4* carriers (“treatment” group) vs. (0) *APOE4* non-carriers (control group). The longevity-related outcome, *Survival85*, was a binary variable defined as follows: (1) survival to age 85 years and above vs. (0) death before age 85. Two mediator variables characterizing weight were considered. The first variable, *AgeMaxW*, was defined as (1) age at the maximum weight ≥75 years vs. (0) age at the maximum weight <75 years and age at the last follow-up or death >80 years. The second variable, *SlopeW*, was defined as (1) relative weight change above the median (tend to have more stable weight during ages 65–80) vs. (0) relative weight change below the median (tend to be losing weight during ages 65–80). *SlopeW* was calculated as (mean value at ages 75–79 − mean value at ages 65–74)/ (mean value at ages 65–74) and dichotomized among individuals with the age at the last follow-up or death >80 years.

The SAS procedure PROC CAUSALMED (SAS Institute Inc, 2021) with the SAS software application version 9.4 was used for the entire analysis. As the outcome and mediators were binary variables, logistic regression models were used for the CMA. The trust-region optimization technique (“TRUREG” option) was used to obtain the maximum likelihood estimates. The codes used to generate the reported output are available upon request from the corresponding author.

For our CMA approach, we focused primarily on three causal effect estimates: total effect (TE), natural indirect effect (NIE), and percentage mediated (PM). In general, TE, a combination of both the direct and indirect effects, provides an estimate of the “treatment” effect on the outcome at the various mediator levels. Here, this estimate compares the chances of survival for *APOE4* carriers vs. non-carriers while taking into account the effect of the mediator. NIE provides further details about the effects of the “treatment” through the mediator by holding the treatment constant and considering the effect of the mediator only. In this analysis, this estimate will compare the chances of survival for the allele carriers at each of the two mediator levels. PM gives the percentage of the total effect that is mediated by the mediator. Other reported estimates include the natural direct effect (NDE) and controlled direct effect (CDE). Although both of these estimates are useful, they were not the focus of this study as they hold the effect of the mediator constant, i.e., these estimates compare the chances of survival for the two “treatment” groups (carriers vs. non-carriers) without considering the effect of the mediator (SAS Institute Inc, 2021).

The CMA approach has four assumptions (Vander and Tyler, 2016): control must be made for (Assumption 1) exposure-outcome confounding, (Assumption 2) mediator-outcome confounding, and (Assumption 3) exposure-mediator confounding, as well as (Assumption 4) no mediator-outcome confounders being affected by the treatment. The following covariates were included in both the mediator and outcome models in order to control for confounding: smoking status (coded 0–never smoked and 1–ever smoked), education (coded 0–less than high school, 1–high school only, and 2–higher education), occurrence of cancer (coded 0–no cancer and 1–cancer), sex (coded 1–male and 2–female), and race (coded 1–White, 2–Black, and 3–Other). Furthermore, we followed A Guideline for Reporting Mediation Analyses (AGReMA) long-form checklist (Lee et al., 2021) (see Supplementary Table S1). See VanderWeele (2015) for additional details and methods.

In addition to reporting results for the total analytic sample, we provided stratified results by sex and race. Instead of creating sex and race subsamples, the “Evaluate” statement was used within PROC CAUSALMED. This allows users to specify covariate levels (such as sex and race) and evaluate the respective conditional causal effects. The “Evaluate” statement uses the entire analytic sample and estimates from the associated model to compute the mean of the specified covariate level(s). SAS documentation on PROC CAUSALMED provides further details (SAS Institute Inc, 2021). Additionally, we only used observations for which all variables involved in either of the models were non-missing (i.e., only those observations with information for “treatment,” outcome, mediator, and all covariates were used).

**FIGURE 2:**
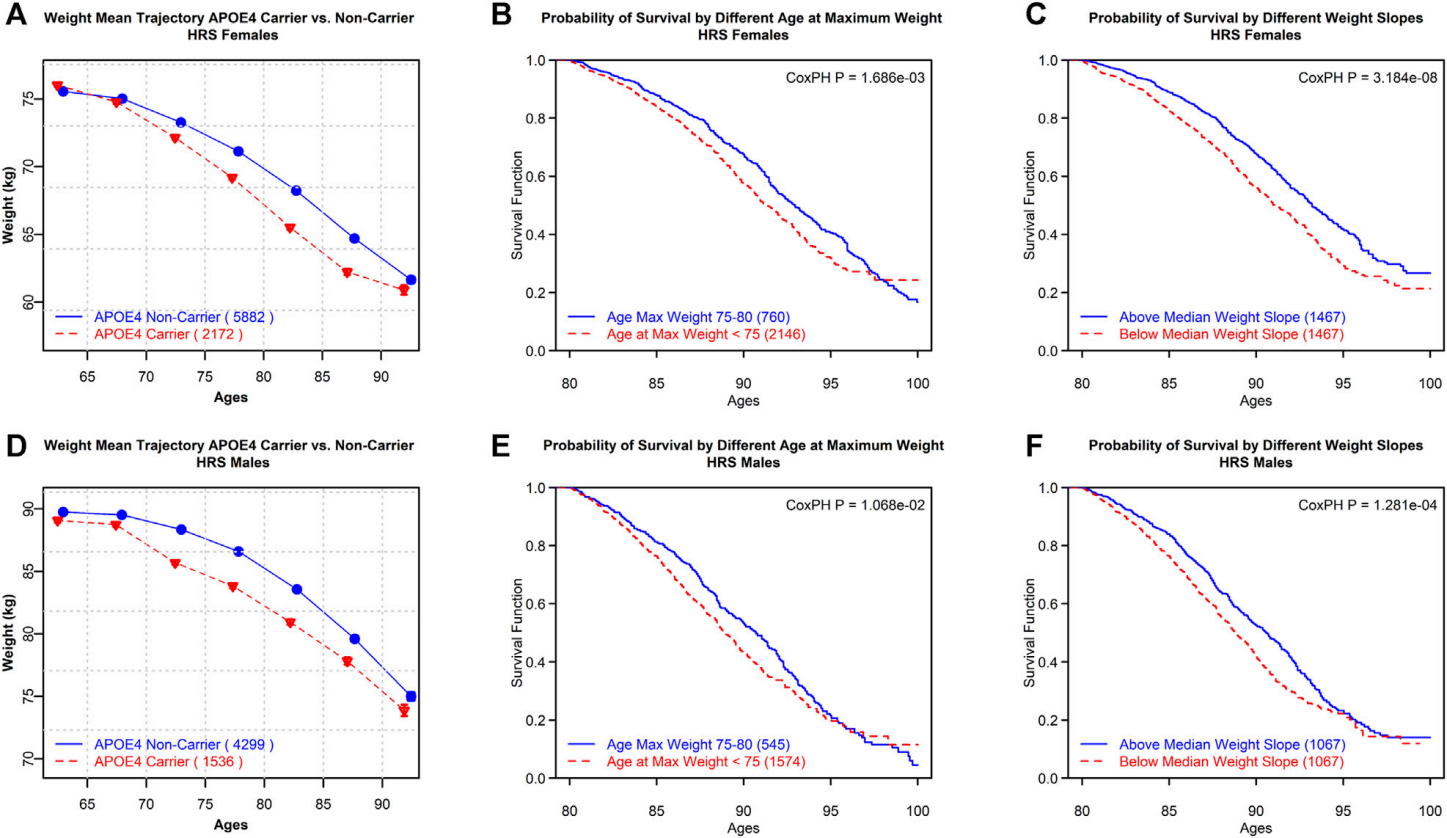
HRS females: **(A)** mean trajectories of body weight in *APOE4* carriers vs. non-carriers. **(B)** Survival trajectories by different ages at reaching maximum values of weight. **(C)** Survival trajectories by different weight slopes. HRS males: **(D)** mean trajectories of body weight in *APOE4* carriers vs. non-carriers. **(E)** Survival trajectories by different age categories at reaching maximum values of weight. **(F)** Survival trajectories by different weight slopes. Cox proportional hazards *p*-value (CoxPH P) is provided as a measure of the statistical significance of differences between survival curves in the respective groups.

Furthermore, using the HRS data, we compared the mean trajectories of the weight for *APOE4* carriers and non-carriers and the survival trajectories by different weight slopes and ages at reaching the maximum values of weight (Figure 2). These variables are proxies for the respective variables defined for BMI because height does not generally demonstrate substantial changes in individuals at ages over 80. Furthermore, a Cox proportional hazards *p*-value is provided on each plot as a measure of the significance of survival models. Cancer is included as a covariate in the Cox proportional hazards *p*-value.

Since the ε2 allele of the *APOE* gene is known for its beneficial effects on longevity (Wolters et al., 2019), we also conducted a sensitivity analysis with this allele removed. In other words, we compared those with the ε3/ε3 genotype (non-carriers) against those with ε3/ε4 or ε4/ε4 genotypes (carriers).

## 3 Results

The mean trajectories of the weight for *APOE4* carriers and non-carriers, as well as survival trajectories by different weight slopes and different ages at reaching maximum values of the weight, are shown in Figure 2. These variables are proxies for similarly defined BMI variables as the height does not show substantial changes for those aged 80+. *APOE4* carriers decline faster in weight with age compared to non-carriers (see Figures 2A and D). Survival was better for individuals with a less steep weight slope and older age at reaching the maximum values of weight (after age 75 vs. less than 75 years) (see Figures 2B, C, E, and F). One may notice the convergence in Figures 2B and E; this convergence is not unexpected. Although the HRS sample itself is not small, the number of people available at ages 95+ is substantially reduced. Furthermore, we tested if there was a significant difference between the incidences of comorbidities among the groups in all the plots in Figure 2. We found that there was no significant difference between the occurrences of cancer or cardiovascular disease among any of the groups.

***AgeMaxW*** (below 75 years) was a significant mediator of the effects of the *APOE* alleles on survival to ages 85+ in the total sample and in samples stratified by sex and race. This was supported by the significant *p*-values for TE and NIE (see Table 3). TE estimates suggest that ε4 carriers are 19%–21% (*p* = 0.020–0.039) more likely to die before age 85 compared to non-carriers. If we hold the “treatment” constant and consider only the mediator effect, then *APOE4* carriers who have a younger *AgeMaxW* are about 2.5% (*p* = 0.026–0.028) more likely to die before age 85 than the carriers who have an older *AgeMaxW*. The percentage of the total effect mediated by the *AgeMaxW* was around 14% for the overall sample and all strata, and while the PM was not statistically significant, it was marginally significant with *p* = 0.074–0.075.

**TABLE 3:** Results of CMA for *APOE4*’s effect on survival up to age 85+ mediated by *AgeMaxW* and *SlopeW*. Results evaluated at different strata. [TE (LC, UC), pval]: [total effect estimate (lower 95% confidence interval and upper 95% confidence interval), *p*-value of total effect estimate], similarly for NIE (natural indirect effect), PM (percentage mediated), CDE (controlled direct effect), and NDE (natural direct effect). TE compares the chances of survival for *APOE4* carriers vs. non-carriers while taking into account the effect of the mediator. NIE holds the treatment constant and considers the effect of the mediator only. PM gives the percentage of the total effect that is mediated by the mediator. NDE and CDE compare the chances of survival for the two “treatment” groups (carriers vs. non-carriers) without considering the effect of the mediator.

Mediator	Treatment	Strata	[TE (LC, UC), pval]	[NIE (LC, UC), pval]	[PM (LC, UC), pval]	[CDE (LC, UC), pval]	[NDE (LC, UC), pval]	N
*AgeMaxW*	*APOE4*	Total	[1.213 (1.034, 1.391), 0.020]*	[1.026 (1.003, 1.049), 0.028]*	[14.244 (−1.382, 29.870), 0.074]	[1.468 (1.034, 1.391), 0.020]*	[1.183 (1.007, 1.358), 0.042]*	3,287
*AgeMaxW*	*APOE4*	Male	[1.207 (1.029, 1.384), 0.023]*	[1.025 (1.003, 1.048), 0.027]*	[14.365 (−1.374, 30.104), 0.074]	[1.468 (1.034, 1.391), 0.020]*	[1.177 (1.003, 1.351), 0.046]*	3,287
*AgeMaxW*	*APOE4*	Female	[1.211 (1.032, 1.390), 0.021]*	[1.025 (1.003, 1.048), 0.027]*	[14.283 (−1.380, 29.946), 0.074]	[1.468 (1.034, 1.391), 0.020]*	[1.181 (1.006, 1.356), 0.043]*	3,287
*AgeMaxW*	*APOE4*	White	[1.211 (1.032, 1.390), 0.021]*	[1.026 (1.003, 1.048), 0.028]*	[14.283 (−1.380, 29.946), 0.074]	[1.468 (1.034, 1.391), 0.020]*	[1.181 (1.006, 1.356), 0.043]*	3,287
*AgeMaxW*	*APOE4*	Black	[1.185 (1.010, 1.361), 0.039]*	[1.023 (1.003, 1.044), 0.026]*	[14.619 (−1.474, 30.713), 0.075]	[1.468 (1.034, 1.391), 0.020]*	[1.158 (0.988, 1.329), 0.069]	3,287
*SlopeW*	*APOE4*	Total	[1.222 (1.034, 1.410), 0.021]*	[1.036 (1.007, 1.065), 0.015]*	[19.102 (−0.502, 38.705), 0.056]	[1.256 (0.934, 1.578), 0.119]	[1.180 (0.995, 1.363), 0.055]	3,020
*SlopeW*	*APOE4*	Male	[1.221 (1.033, 1.409), 0.021]*	[1.036 (1.007, 1.065), 0.015]*	[19.122 (−0.437, 38.682), 0.055]	[1.256 (0.934, 1.578), 0.119]	[1.179 (0.996, 1.362), 0.056]	3,020
*SlopeW*	*APOE4*	Female	[1.221 (1.033, 1.408), 0.021]*	[1.036 (1.007, 1.064), 0.014]*	[19.135 (−0.389, 38.659), 0.055]	[1.256 (0.934, 1.577), 0.119]	[1.178 (0.995, 1.361), 0.056]	3,020
*SlopeW*	*APOE4*	White	[1.221 (1.033, 1.408), 0.021]*	[1.036 (1.007, 1.064), 0.014]*	[19.135 (−0.389, 38.659), 0.055]	[1.256 (0.934, 1.577), 0.119]	[1.178 (0.995, 1.361), 0.056]	3,020
*SlopeW*	*APOE4*	Black	[1.221 (1.033, 1.408), 0.021]*	[1.036 (1.007, 1.065), 0.014]*	[19.131 (−0.401, 38.663), 0.055]	[1.256 (0.934, 1.577), 0.119]	[1.178 (0.995, 1.362), 0.056]	3,020

Effect estimates are presented on the relative risk scale. *, **s**ignificant *p*-value (<0.05); N, number of observations used.

***SlopeW*** was also a significant mediator of the *APOE4* effects on survival for 85+ years in the total sample, with about a 4% higher (NIE: *p*-value = 0.014–0.015) (see Table 3) chance of death before age 85 when holding the “treatment” constant and considering the mediator’s effect only. TE estimates suggested that the chances of dying before age 85 were about 22% higher for *APOE4* carriers vs. non-carriers (*p*-value = 0.021). A very similar effect was seen at all strata levels with similar *p*-values. The percentage of the total effect mediated by *SlopeW*, around 19%, was marginally significant (*p* = 0.055–0.056).

In our sensitivity analysis, which has the same framework as the initial analysis but with ε2 carriers removed from the *APOE4* “treatment,” results of both *AgeMaxW* and *SlopeW* are comparable to the initial results and are available in Supplementary Table S2. It is worth noting that these results are “improved” slightly, in terms of the estimated size and significance for TE and NIE, and are similar in size and effect for other estimates.

## 4 Discussion

Findings of this CMA suggest that dynamics of aging-related changes in body weight mediates the effect of *APOE4* on longevity. Specifically, *APOE4* carriers have lower chances of survival to age 85+, and estimates suggest that 14%–19% of this association may be related to reaching the maximum weight at earlier ages. The *APOE4* carriers were about 19%–22% more likely to die before age 85, compared to non-carriers, when considering the mediating effects of *SlopeW* and *AgeMaxW*. To the best of our knowledge, no studies have yet considered the patterns of aging-related changes in body weight over the life course as mediators of the negative association between *APOE4* and longevity.

One may argue that the well-known beneficial effects of the ε2 allele of the *APOE* gene (Wolters et al., 2019) may contribute to the difference in survival chances seen in this CMA. To account for this, and to ensure that *APOE4’*s effect on longevity is indeed mediated by aging-related changes in body weight, we conducted a sensitivity analysis with the ε2 allele carriers removed from the “treatment” variable. This increased the effect size and significance of most TE and NIE estimates (Supplementary Table S2). Other estimates are relatively similar in effect size and significance, although they still vary from the original CMA results. This further suggests that the effect of *APOE4* on longevity is partially mediated by aging-related changes in body weight, and the ε2 allele is not the source of the difference in survival chances seen in this CMA.

A prominent mechanism involved in many aging and longevity studies is lipids/cardiovascular disease(s). We did not consider lipids as a confounding factor as earlier studies (Kulminski et al., 2014; Tejedor et al., 2014; Loika et al., 2022; Ozen et al., 2022) do not support the connection between APOE and BMI through lipids nor cardiovascular disease. Furthermore, since cancer is a disease known to cause weight loss, we included cancer as a covariate to our analysis.

One should emphasize that the CMA that was implemented in this study has advantages over TMA. Since TMA effects are based on the coefficients of regression models, it is bound by parametric assumptions, whereas the natural effects from CMA are not. Additionally, TMA does not usually include an interaction between the treatment and mediator (Rijnhart et al., 2021). Another advantage is the use of a causal inference approach, which utilizes a counterfactual framework. Although TMA is a useful tool for detecting mediating effects and describing the total, direct, and indirect effects, CMA breaks these effects down even more, helping to better understand the underlying mechanism of the negative association between *APOE4* and longevity.

Our results are in line with earlier studies that demonstrated a detrimental effect of *APOE4* on longevity (Arbeev et al., 2011; Kulminski et al., 2011; Arbeev et al., 2012; Kulminski et al., 2014; Garatachea et al., 2015; Zeng et al., 2016; Yashin et al., 2018a; Yashin et al., 2018b; Abondio et al., 2019; Gurinovich et al., 2019; Kulminski et al., 2019; Santovito, Galli, and Ruberto, 2019; Sebastiani et al., 2019; Kulminski et al., 2022). They also suggested that *APOE4* facilitates physical aging (manifested in an earlier and faster decline in weight) (Yashin et al., 2010), which, in turn, can causally contribute to reduced longevity. Previously, we showed that the longest-lived individuals reach peak values of weight/BMI and start to decline later in their lives compared to people with a conventional lifespan (Yashin et al., 2013; Yashin et al., 2016). The ability to grow in size and postpone weight loss until older ages may also correlate with better physical resilience to life stressors, which is essential for survival at the oldest ages (Ukraintseva et al., 2021).

A potential limitation to this study is that, while highly relevant covariates were included in both models (smoking status, education level, sex, and race), there still may exist unmeasured confounders, creating the potential for biased effect estimates. This CMA also revealed that *AgeMaxW* and *SlopeW* are factors that may explain only a part (albeit substantial) of the negative association between *APOE4* and longevity because they did not meet the criteria to satisfy “complete mediation” ( PM≥80% ) (Kenny, 2021). This suggests that there are other causal factors to be discovered, which warrants further investigation of the mechanisms involved in the association between *APOE4* and longevity using other mediators, such as those involved in the dysregulations of cholesterol transport, impaired myelination, hypometabolism, and other traits associated with *APOE4* that may also affect longevity (Blanchard et al., 2022; Zhang et al., 2023). We note that we did not correct for multiple comparisons in our analyses because our mediator variables are substantially correlated [0.54, 95% CI: (0.52, 0.56)]; therefore, applying the Bonferroni correction would be too conservative here. Nevertheless, the results with significance close to 0.05 (such as NIE for *AgeMaxW*) should be interpreted with care. Additionally, the PM results did not reach the standard statistical significance (*p* < 0.05), but they did reach marginal significance (*p* = 0.055–0.075), so replication in larger studies is needed to verify the finding.

In conclusion, this causal mediation analysis found that *APOE4* carriers have lower chances of surviving to age 85 and beyond, in part, because they reach peak values of body weight at younger ages and decline faster afterward compared to non-carriers. Postponing and attenuating the decline in weight in *APOE4* carriers could be a promising target of pro-longevity interventions.

## Data Availability

The data analyzed in this study are subject to the following licenses/restrictions: dbGaP and the University of Michigan Data Use Certification Agreement restrictions. Requests to access these datasets should be directed to the RAND HRS data provider RANDHRSHelp@rand.org. Access to the HRS genetic data is provided by the database of Genotypes and Phenotypes, dbGaP (Study accession: phs000428. v2. p2).
